# 

**Published:** 1892-10

**Authors:** 


10 cts. a copy. OCTOBER, 1892. $1.00 per year.

### HALL'S JOURNAL of HEALTH

ESTABLISHED 1854

UPTOWN OFFICE, 340 WEST 59th STREET.

Entered as Second-Class Matter at the Post-Office, New York.

